# Supplementary material for: A Humanized Yeast Phenomic Model of Deoxycytidine Kinase to Predict Genetic Buffering of Nucleoside Analog Cytotoxicity
Source: Genes (Basel). 2019 Sep 30;10(10):770. doi: 10.3390/genes10100770 (PMC6826991; doi:10.3390/genes10100770)
Supplement: Supplementary file 1 [file genes-10-00770-s001.zip › Additional_Files/Additional_File7_GOTermHeatmaps.zip!/Additional_File7_GOTermHeatmaps/rRNA_metabolic_process.pdf]

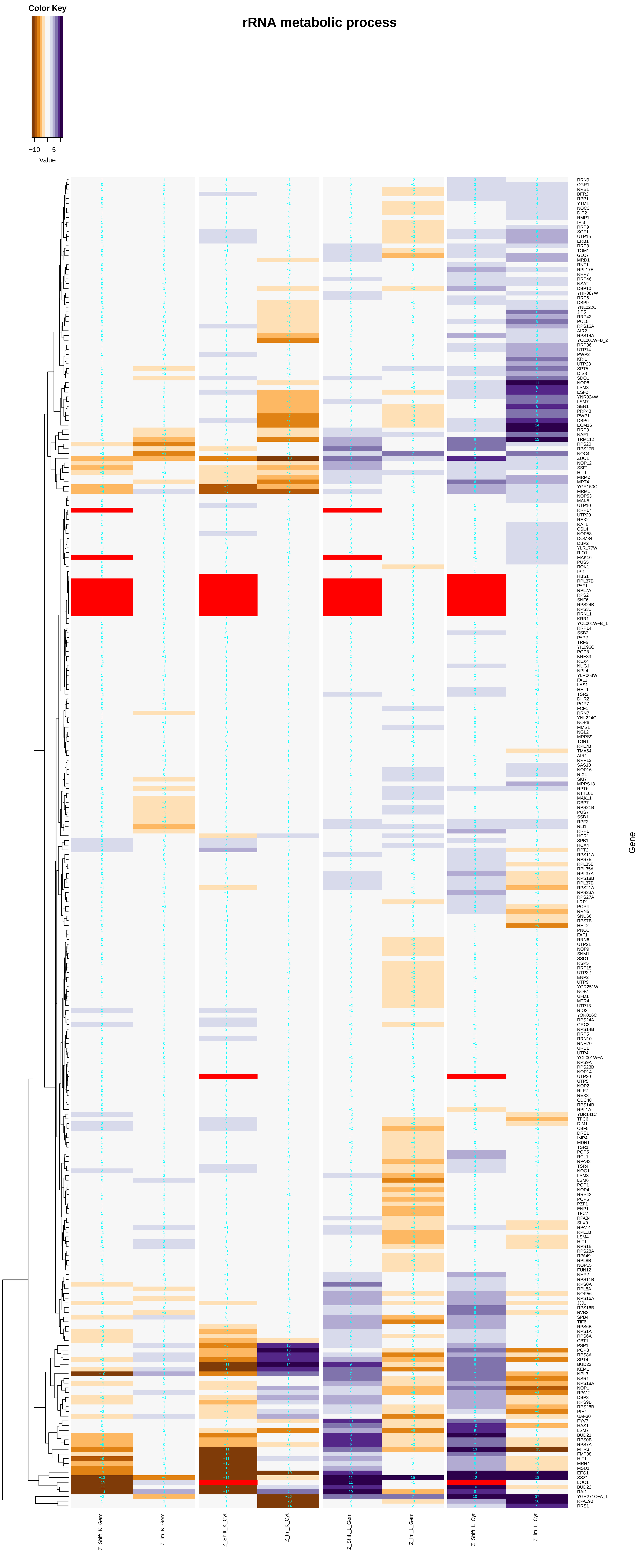

rRNA metabolic process
Color Key
−10
5
Value
Gene
Z_Shift_K_Gem
Z_Im_K_Gem
Z_Shift_K_Cyt
Z_Im_K_Cyt
Z_Shift_L_Gem
Z_Im_L_Gem
Z_Shift_L_Cyt
Z_Im_L_Cyt

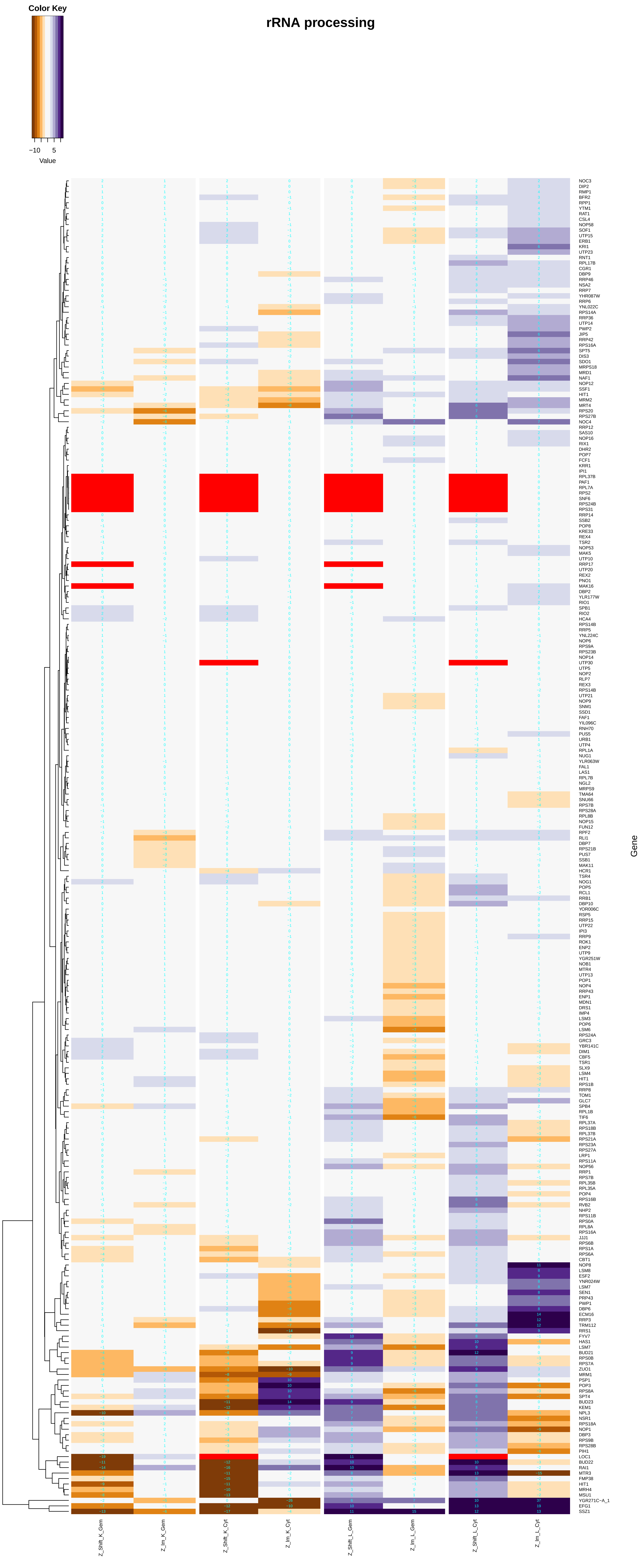
rRNA processing
Color Key
−10
5
Value
Z_Shift_K_Gem
Z_Im_K_Gem
Z_Shift_K_Cyt
Z_Im_K_Cyt
Z_Shift_L_Gem
Z_Im_L_Gem
Z_Shift_L_Cyt
Z_Im_L_Cyt
Gene

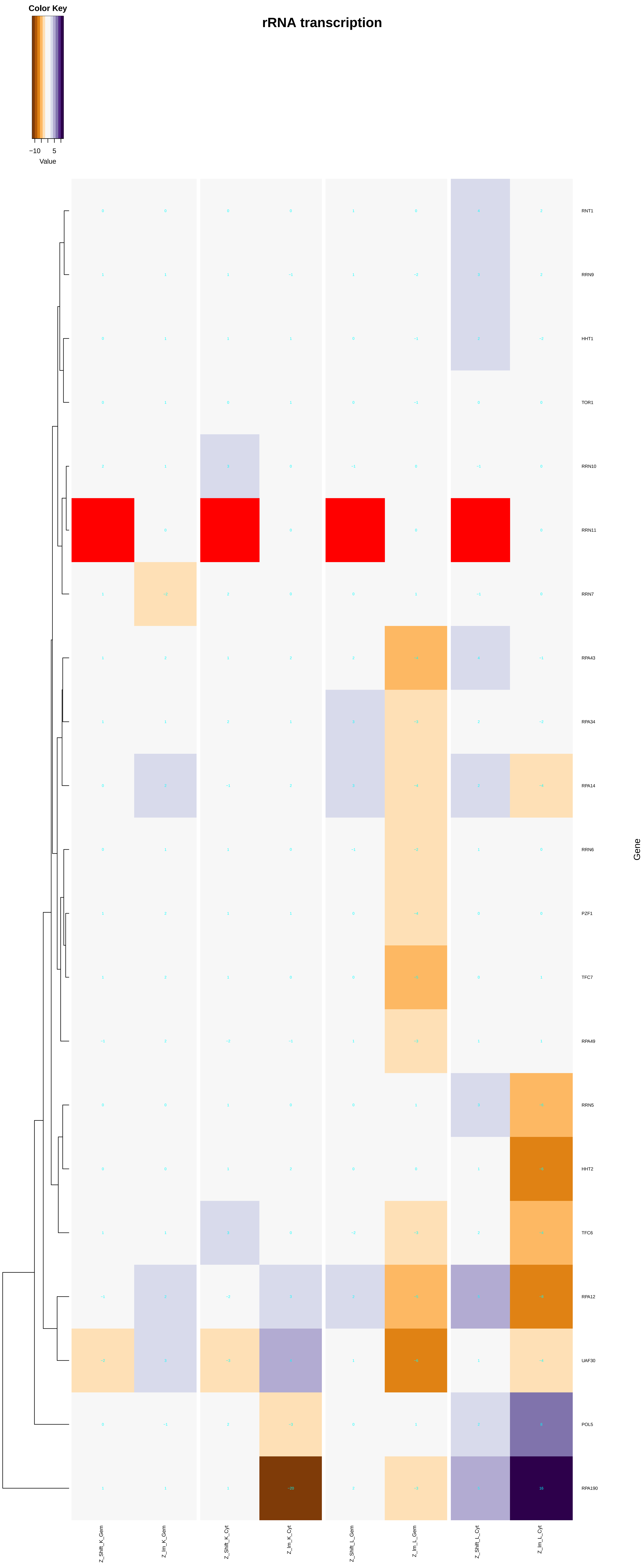

Color Key
−10
5
Value
rRNA transcription
RNT1
RRN9
HHT1
TOR1
RRN10
RRN11
RRN7
RPA43
RPA34
RPA14
RRN6
PZF1
TFC7
RPA49
RRN5
HHT2
TFC6
RPA12
UAF30
POL5
RPA190
Gene
Z_Shift_K_Gem
Z_Im_K_Gem
Z_Shift_K_Cyt
Z_Im_K_Cyt
Z_Shift_L_Gem
Z_Im_L_Gem
Z_Shift_L_Cyt
Z_Im_L_Cyt

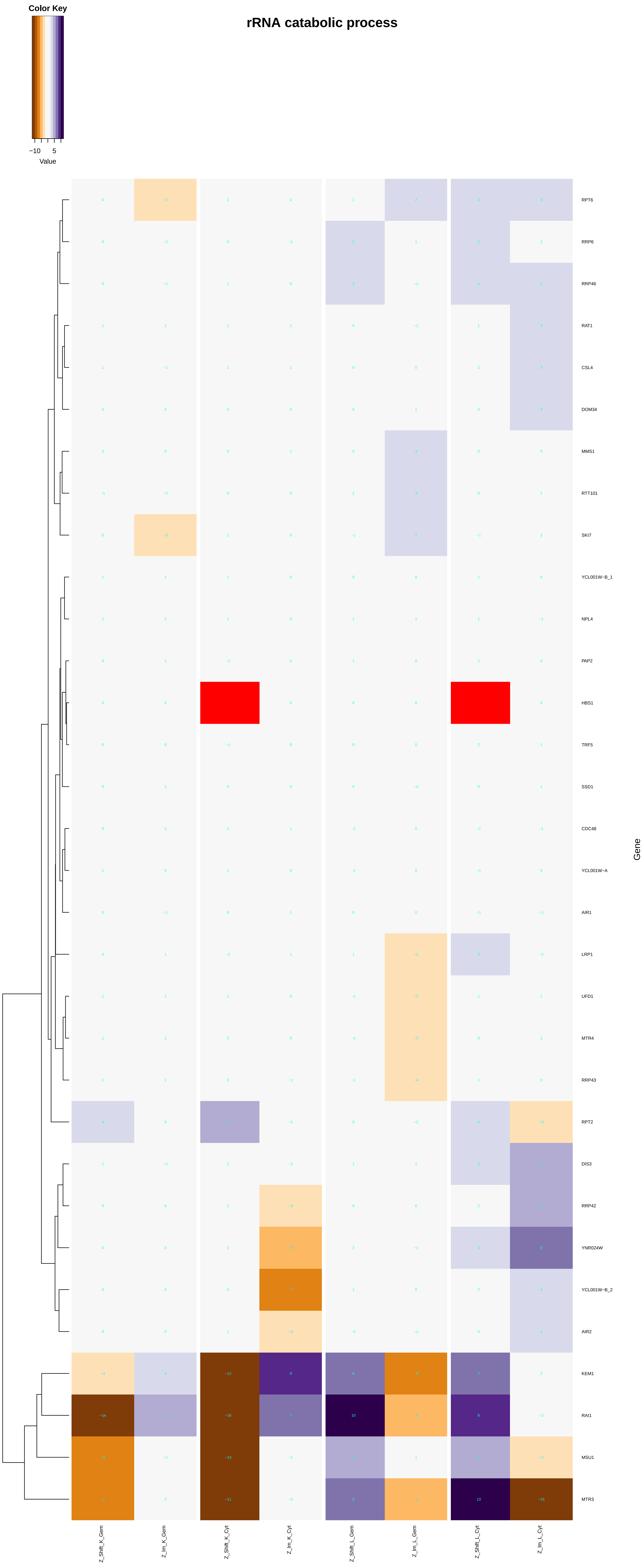

rRNA catabolic process
Color Key
−10
5
Value
RPT6
RRP6
RRP46
RAT1
CSL4
DOM34
MMS1
RTT101
SKI7
YCL001W−B_1
NPL4
PAP2
HBS1
TRF5
SSD1
CDC48
YCL001W−A
AIR1
LRP1
UFD1
MTR4
RRP43
RPT2
DIS3
RRP42
YNR024W
YCL001W−B_2
AIR2
KEM1
RAI1
MSU1
MTR3
Gene
Z_Shift_K_Gem
Z_Im_K_Gem
Z_Shift_K_Cyt
Z_Im_K_Cyt
Z_Shift_L_Gem
Z_Im_L_Gem
Z_Shift_L_Cyt
Z_Im_L_Cyt

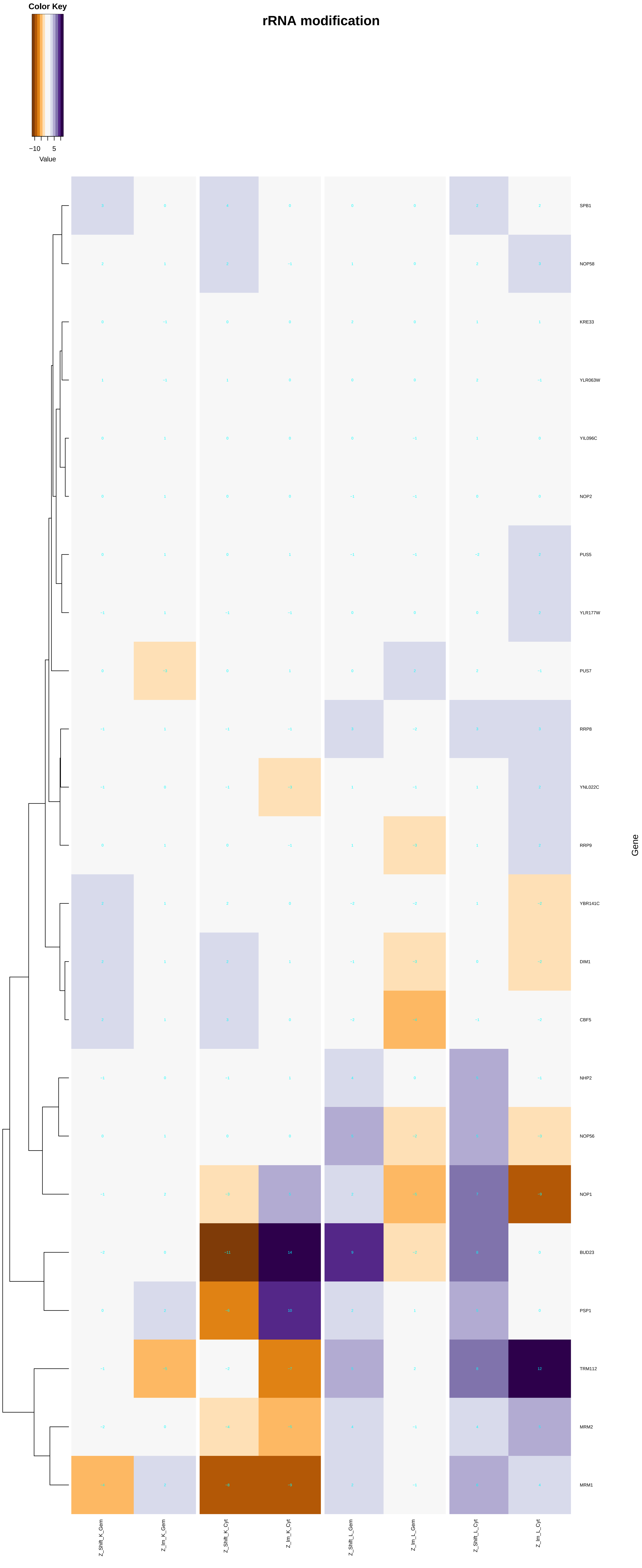

Color Key
−10
5
Value
rRNA modification
SPB1
NOP58
KRE33
YLR063W
YIL096C
NOP2
PUS5
YLR177W
PUS7
RRP8
YNL022C
RRP9
YBR141C
DIM1
CBF5
NHP2
NOP56
NOP1
BUD23
PSP1
TRM112
MRM2
MRM1
Gene
Z_Shift_K_Gem
Z_Im_K_Gem
Z_Shift_K_Cyt
Z_Im_K_Cyt
Z_Shift_L_Gem
Z_Im_L_Gem
Z_Shift_L_Cyt
Z_Im_L_Cyt

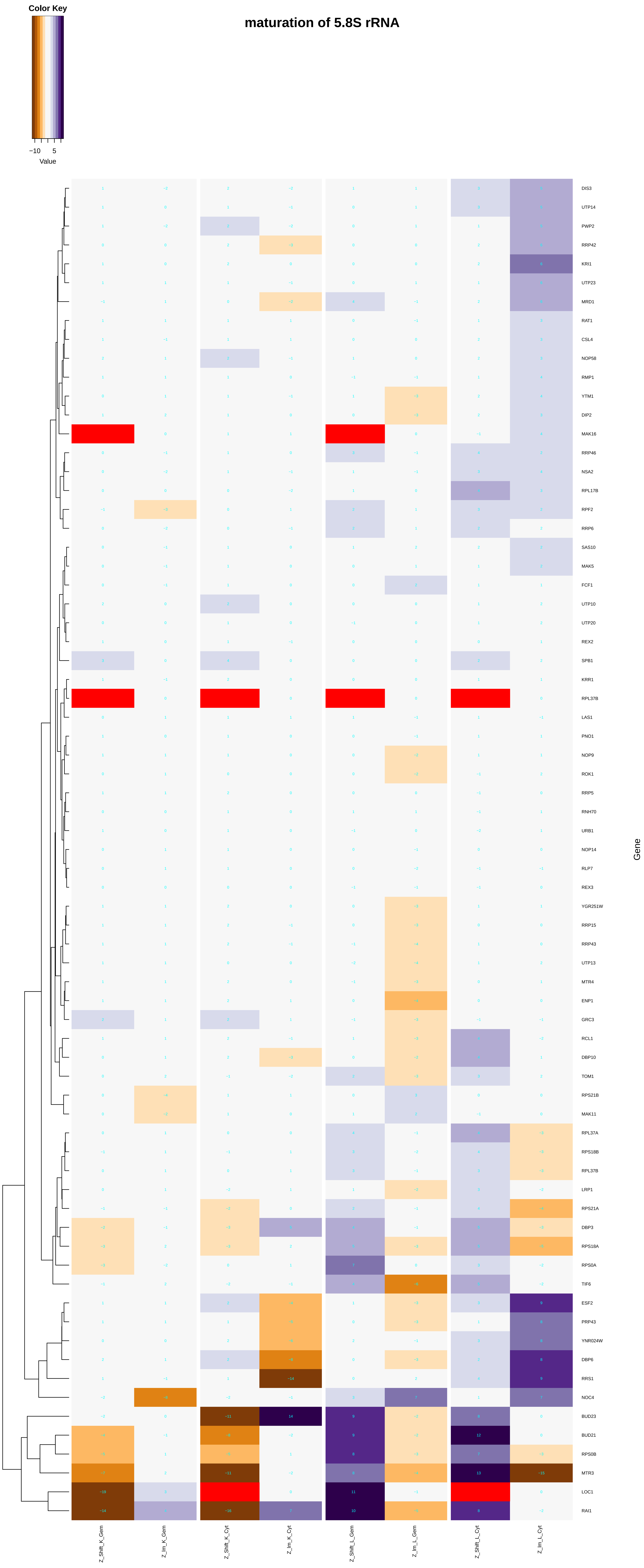

maturation of 5.8S rRNA
Color Key
−10
5
Value
DIS3
UTP14
PWP2
RRP42
KRI1
UTP23
MRD1
RAT1
CSL4
NOP58
RMP1
YTM1
DIP2
MAK16
RRP46
NSA2
RPL17B
RPF2
RRP6
SAS10
MAK5
FCF1
UTP10
UTP20
REX2
SPB1
KRR1
RPL37B
LAS1
PNO1
NOP9
ROK1
RRP5
RNH70
URB1
NOP14
RLP7
REX3
YGR251W
RRP15
RRP43
UTP13
MTR4
ENP1
GRC3
RCL1
DBP10
TOM1
RPS21B
MAK11
RPL37A
RPS18B
RPL37B
LRP1
RPS21A
DBP3
RPS18A
RPS0A
TIF6
ESF2
PRP43
YNR024W
DBP6
RRS1
NOC4
BUD23
BUD21
RPS0B
MTR3
LOC1
RAI1
Gene
Z_Shift_K_Gem
Z_Im_K_Gem
Z_Shift_K_Cyt
Z_Im_K_Cyt
Z_Shift_L_Gem
Z_Im_L_Gem
Z_Shift_L_Cyt
Z_Im_L_Cyt

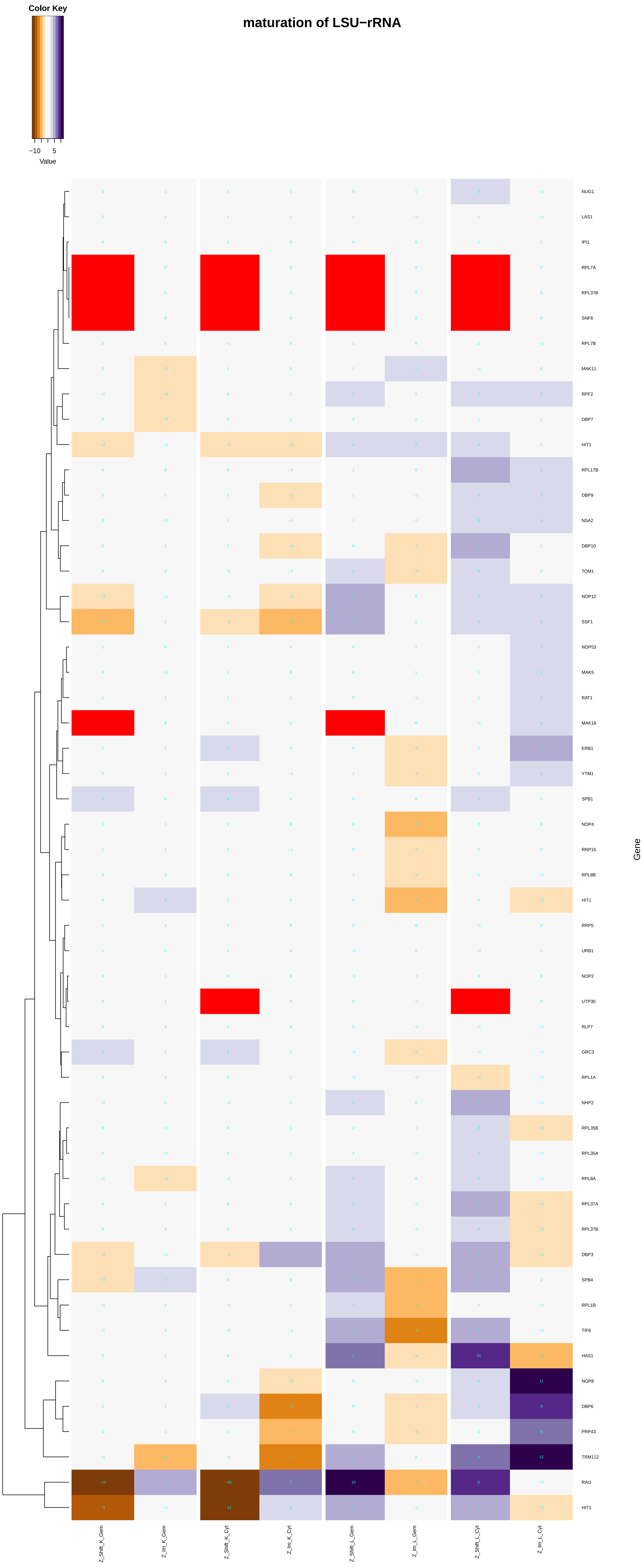
maturation of LSU−rRNA
Color Key
−10
5
Value
NUG1
LAS1
IPI1
RPL7A
RPL37B
SNF6
RPL7B
MAK11
RPF2
DBP7
HIT1
RPL17B
DBP9
NSA2
DBP10
TOM1
NOP12
SSF1
NOP53
MAK5
RAT1
MAK16
ERB1
YTM1
SPB1
NOP4
RRP15
RPL8B
HIT1
RRP5
URB1
NOP2
UTP30
RLP7
GRC3
RPL1A
NHP2
RPL35B
RPL35A
RPL8A
RPL37A
RPL37B
DBP3
SPB4
RPL1B
TIF6
HAS1
NOP8
DBP6
PRP43
TRM112
RAI1
HIT1
Gene
Z_Shift_K_Gem
Z_Im_K_Gem
Z_Shift_K_Cyt
Z_Im_K_Cyt
Z_Shift_L_Gem
Z_Im_L_Gem
Z_Shift_L_Cyt
Z_Im_L_Cyt

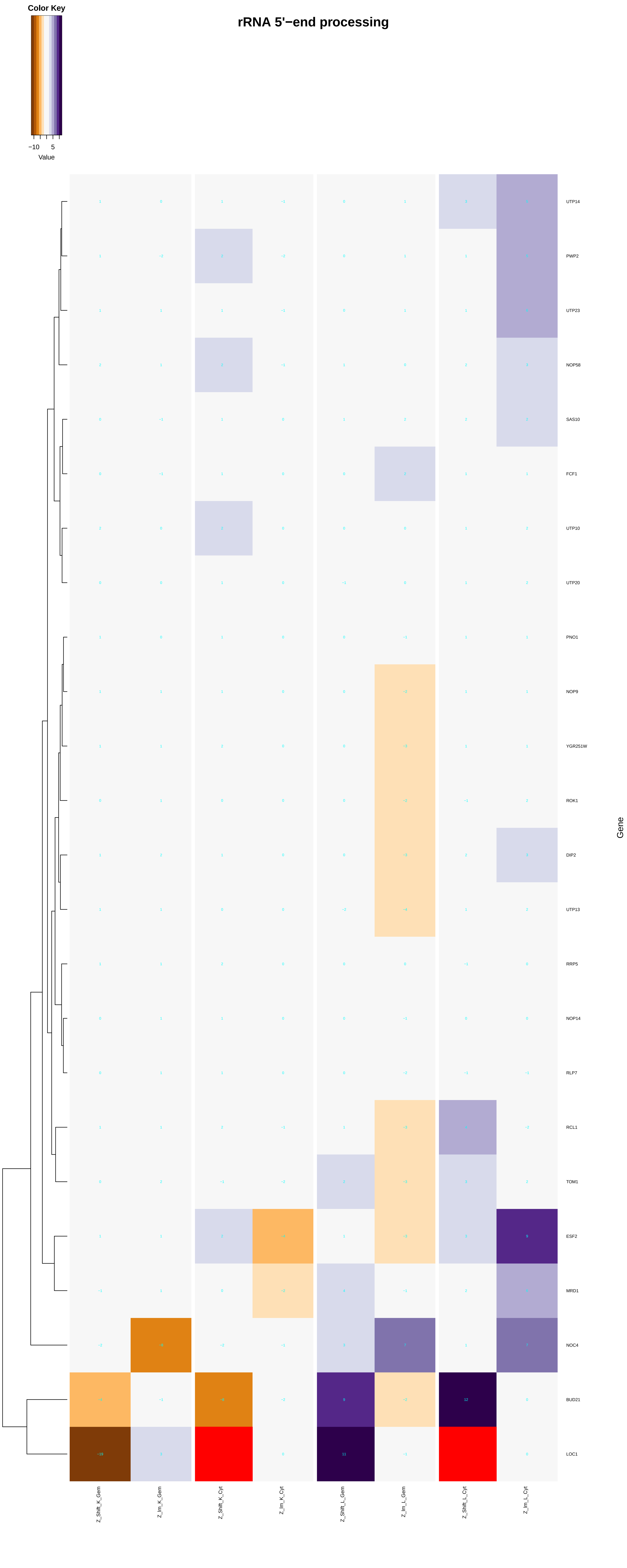
rRNA 5'−end processing
Color Key
−10
5
Value
UTP14
PWP2
UTP23
NOP58
SAS10
FCF1
UTP10
UTP20
PNO1
NOP9
YGR251W
ROK1
DIP2
UTP13
RRP5
NOP14
RLP7
RCL1
TOM1
ESF2
MRD1
NOC4
BUD21
LOC1
Gene
Z_Shift_K_Gem
Z_Im_K_Gem
Z_Shift_K_Cyt
Z_Im_K_Cyt
Z_Shift_L_Gem
Z_Im_L_Gem
Z_Shift_L_Cyt
Z_Im_L_Cyt

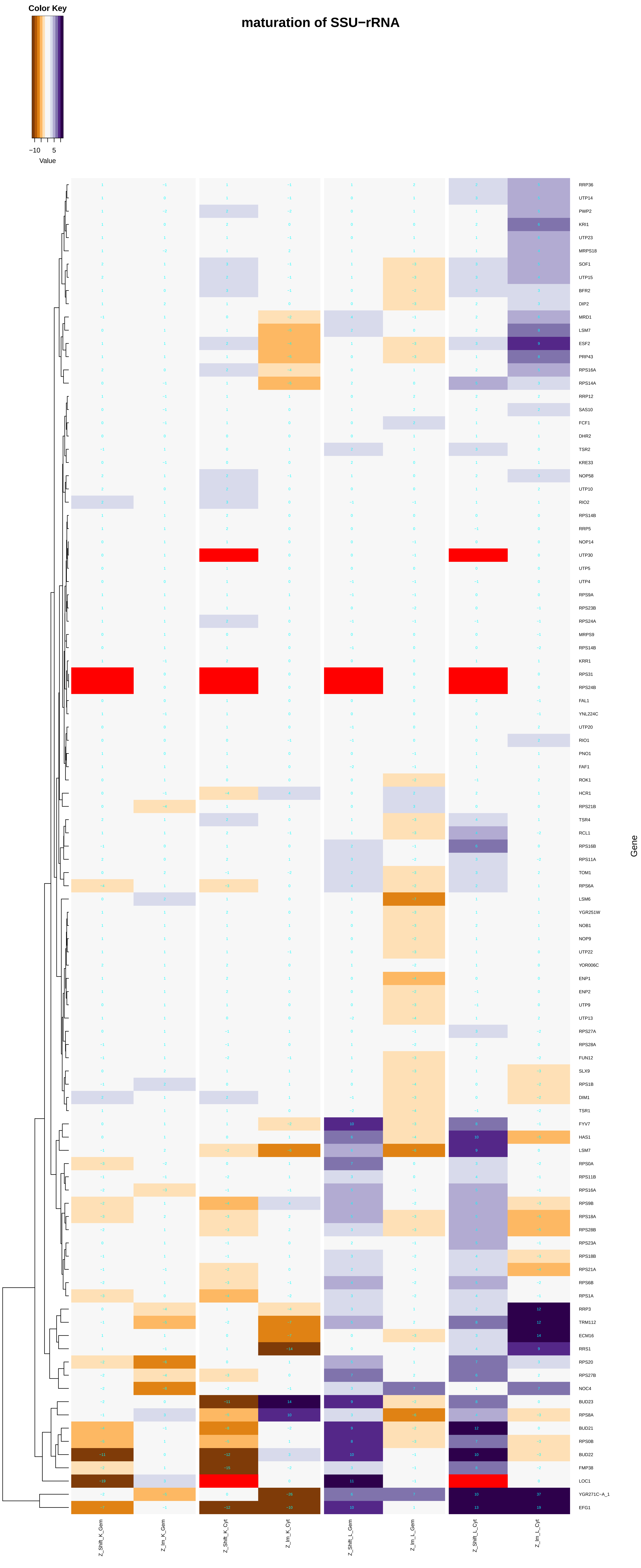
maturation of SSU−rRNA
Color Key
−10
5
Value
RRP36
UTP14
PWP2
KRI1
UTP23
MRPS18
SOF1
UTP15
BFR2
DIP2
MRD1
LSM7
ESF2
PRP43
RPS16A
RPS14A
RRP12
SAS10
FCF1
DHR2
TSR2
KRE33
NOP58
UTP10
RIO2
RPS14B
RRP5
NOP14
UTP30
UTP5
UTP4
RPS9A
RPS23B
RPS24A
MRPS9
RPS14B
KRR1
RPS31
RPS24B
FAL1
YNL224C
UTP20
RIO1
PNO1
FAF1
ROK1
HCR1
RPS21B
TSR4
RCL1
RPS16B
RPS11A
TOM1
RPS6A
LSM6
YGR251W
NOB1
NOP9
UTP22
YOR006C
ENP1
ENP2
UTP9
UTP13
RPS27A
RPS28A
FUN12
SLX9
RPS1B
DIM1
TSR1
FYV7
HAS1
LSM7
RPS0A
RPS11B
RPS16A
RPS9B
RPS18A
RPS28B
RPS23A
RPS18B
RPS21A
RPS6B
RPS1A
RRP3
TRM112
ECM16
RRS1
RPS20
RPS27B
NOC4
BUD23
RPS8A
BUD21
RPS0B
BUD22
FMP38
LOC1
YGR271C−A_1
EFG1
Gene
Z_Shift_K_Gem
Z_Im_K_Gem
Z_Shift_K_Cyt
Z_Im_K_Cyt
Z_Shift_L_Gem
Z_Im_L_Gem
Z_Shift_L_Cyt
Z_Im_L_Cyt

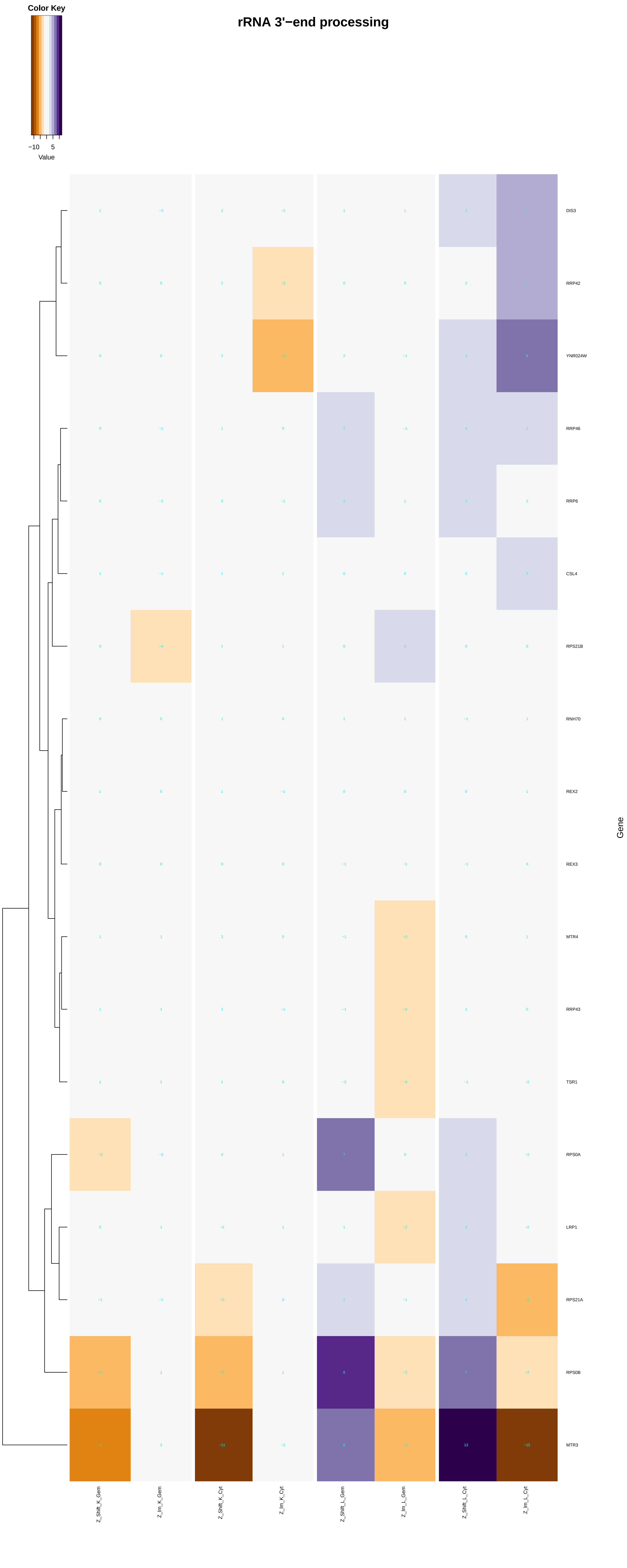
Color Key
−10
5
Value
rRNA 3'−end processing
DIS3
RRP42
YNR024W
RRP46
RRP6
CSL4
RPS21B
RNH70
REX2
REX3
MTR4
RRP43
TSR1
RPS0A
LRP1
RPS21A
RPS0B
MTR3
Gene
Z_Shift_K_Gem
Z_Im_K_Gem
Z_Shift_K_Cyt
Z_Im_K_Cyt
Z_Shift_L_Gem
Z_Im_L_Gem
Z_Shift_L_Cyt
Z_Im_L_Cyt

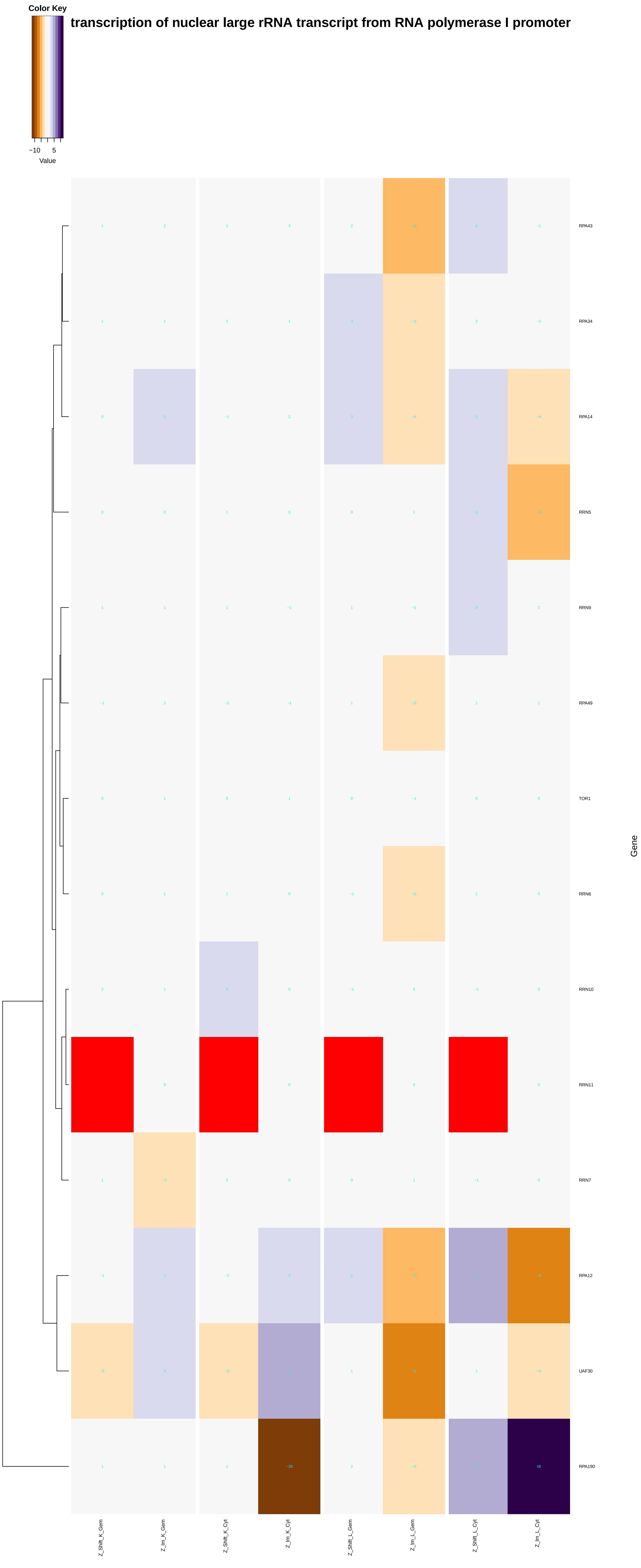

Color Key
−10
5
Value
transcription of nuclear large rRNA transcript from RNA polymerase I promoter
RPA43
RPA34
RPA14
RRN5
RRN9
RPA49
TOR1
RRN6
RRN10
RRN11
RRN7
RPA12
UAF30
RPA190
Gene
Z_Shift_K_Gem
Z_Im_K_Gem
Z_Shift_K_Cyt
Z_Im_K_Cyt
Z_Shift_L_Gem
Z_Im_L_Gem
Z_Shift_L_Cyt
Z_Im_L_Cyt

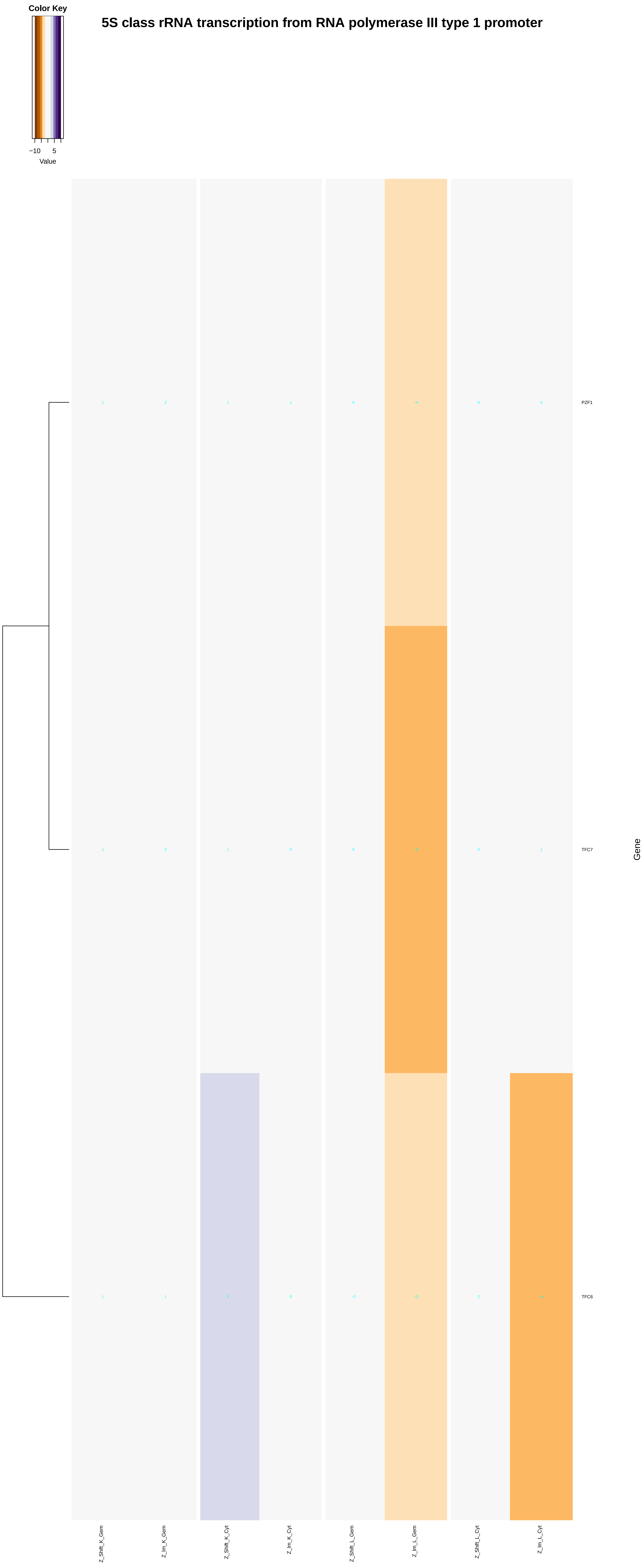
Color Key
−10
5
Value
5S class rRNA transcription from RNA polymerase III type 1 promoter
1
2
1
1
0
−4
0
0
PZF1
1
2
1
0
0
−5
0
1
TFC7
1
1
3
0
−2
−3
2
−4
TFC6
Gene
Z_Shift_K_Gem
Z_Im_K_Gem
Z_Shift_K_Cyt
Z_Im_K_Cyt
Z_Shift_L_Gem
Z_Im_L_Gem
Z_Shift_L_Cyt
Z_Im_L_Cyt

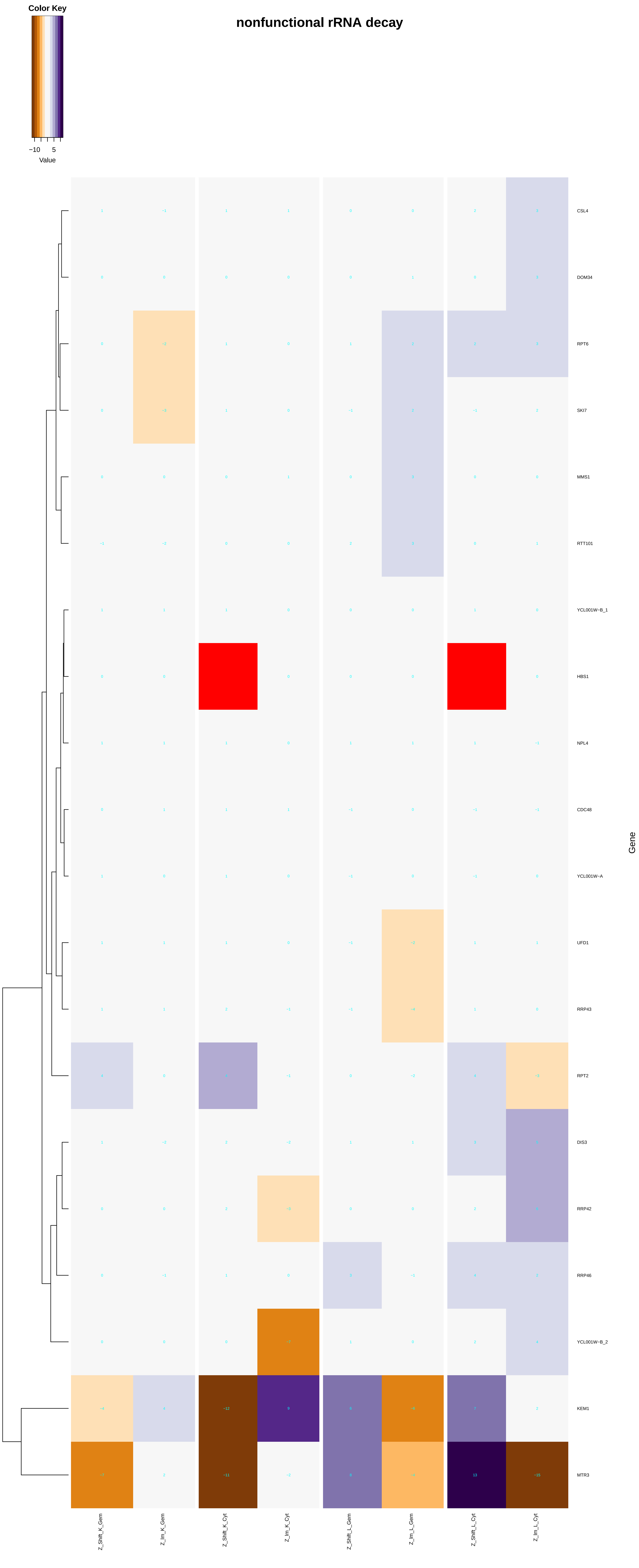
nonfunctional rRNA decay
Color Key
−10
5
Value
CSL4
DOM34
RPT6
SKI7
MMS1
RTT101
YCL001W−B_1
HBS1
NPL4
CDC48
YCL001W−A
UFD1
RRP43
RPT2
DIS3
RRP42
RRP46
YCL001W−B_2
KEM1
MTR3
Gene
Z_Shift_K_Gem
Z_Im_K_Gem
Z_Shift_K_Cyt
Z_Im_K_Cyt
Z_Shift_L_Gem
Z_Im_L_Gem
Z_Shift_L_Cyt
Z_Im_L_Cyt

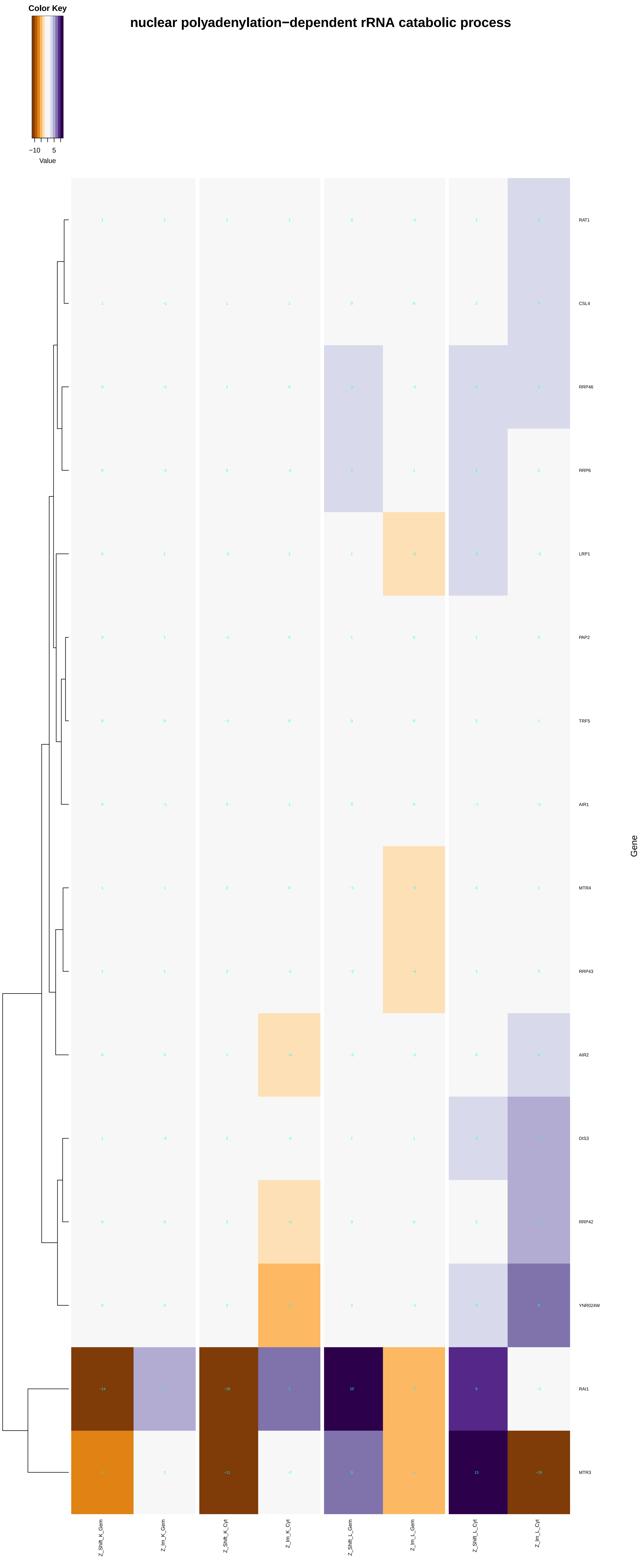

nuclear polyadenylation−dependent rRNA catabolic process
Color Key
−10
5
Value
RAT1
CSL4
RRP46
RRP6
LRP1
PAP2
TRF5
AIR1
MTR4
RRP43
AIR2
DIS3
RRP42
YNR024W
RAI1
MTR3
Gene
Z_Shift_K_Gem
Z_Im_K_Gem
Z_Shift_K_Cyt
Z_Im_K_Cyt
Z_Shift_L_Gem
Z_Im_L_Gem
Z_Shift_L_Cyt
Z_Im_L_Cyt

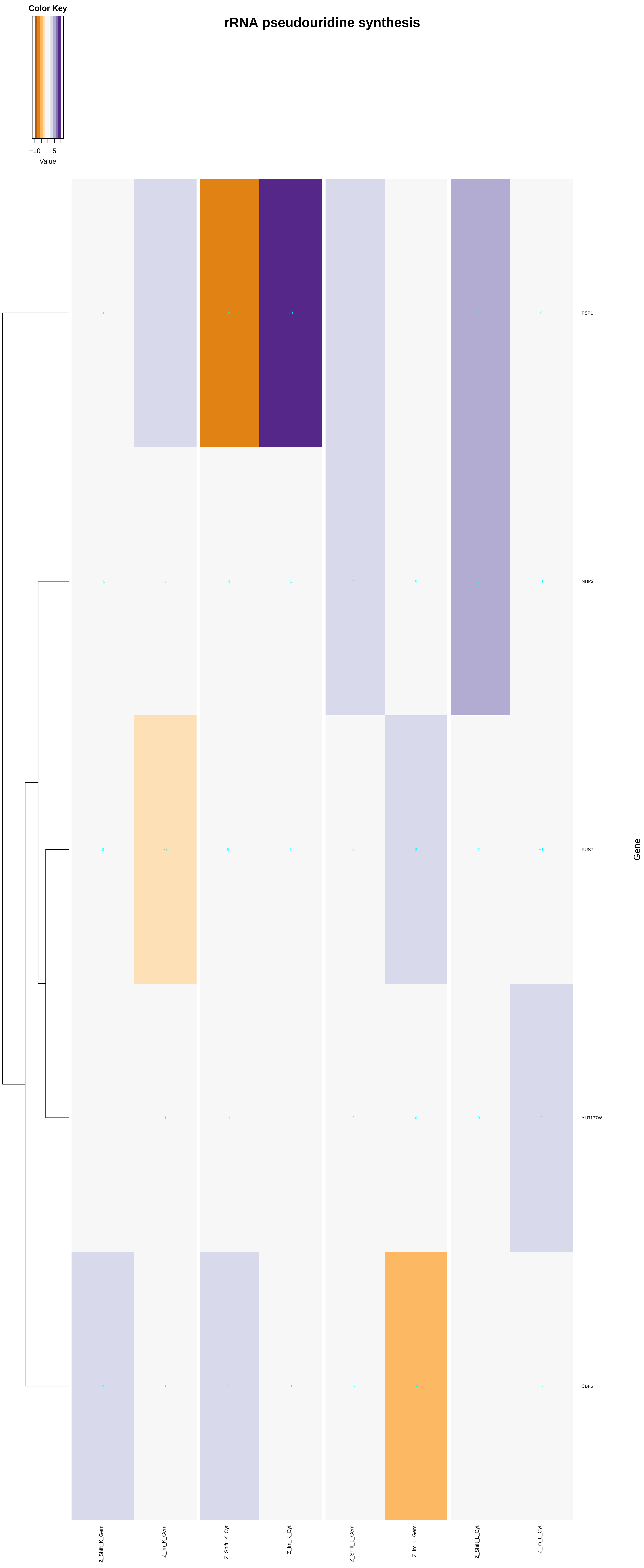

rRNA pseudouridine synthesis
Color Key
−10 5
Value
PSP1
NHP2
PUS7
YLR177W
CBF5
Gene
Z_Shift_K_Gem
Z_lm_K_Gem
Z_Shift_K_Cyt
Z_lm_K_Cyt
Z_Shift_L_Gem
Z_lm_L_Gem
Z_Shift_L_Cyt
Z_lm_L_Cyt

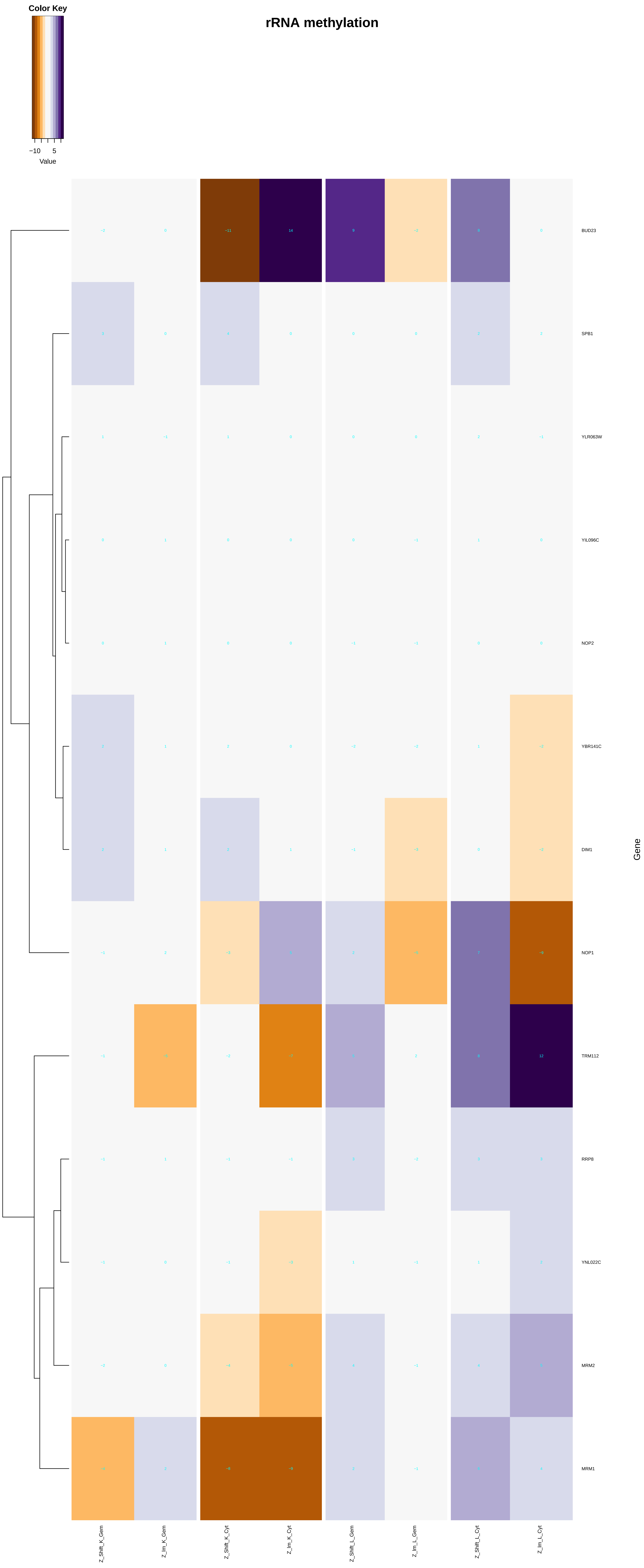
rRNA methylation
Color Key
−10
5
Value
BUD23
SPB1
YLR063W
YIL096C
NOP2
YBR141C
DIM1
NOP1
TRM112
RRP8
YNL022C
MRM2
MRM1
Gene
Z_Shift_K_Gem
Z_Im_K_Gem
Z_Shift_K_Cyt
Z_Im_K_Cyt
Z_Shift_L_Gem
Z_Im_L_Gem
Z_Shift_L_Cyt
Z_Im_L_Cyt

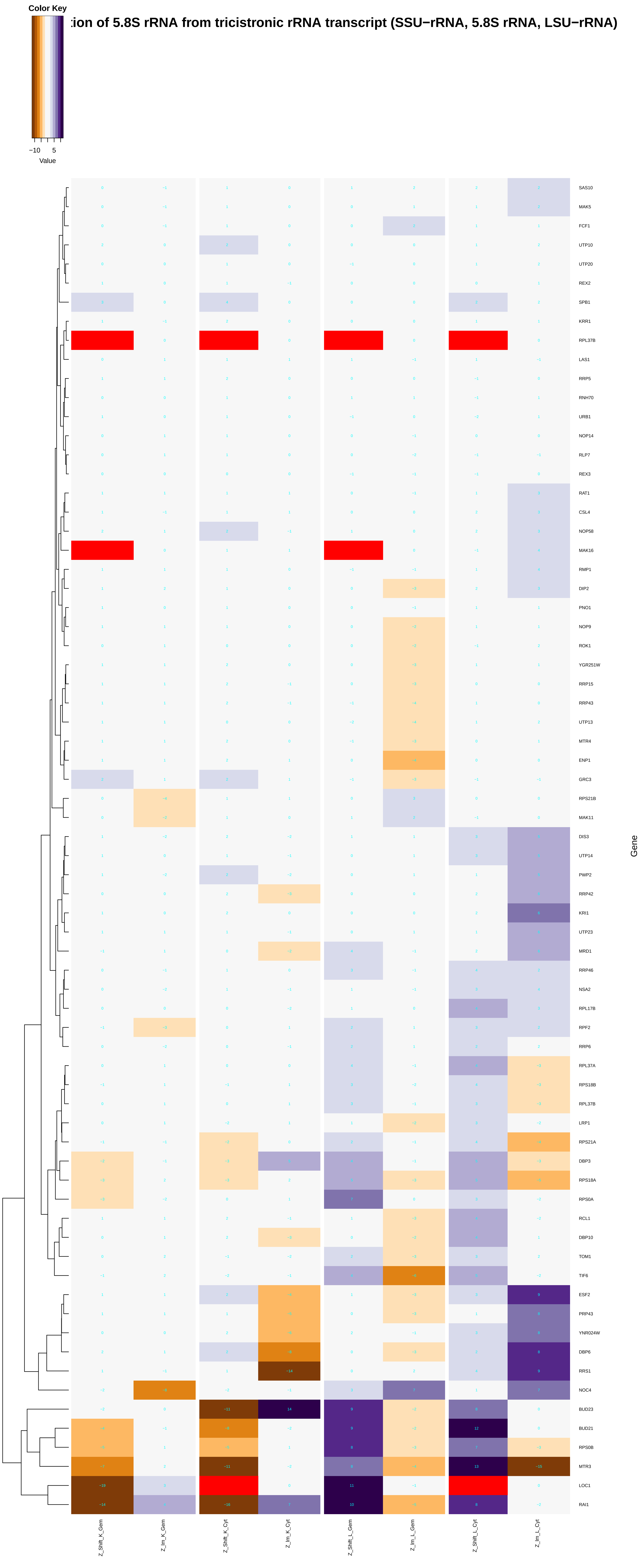

Color Key
−10
5
Value
:ion of 5.8S rRNA from tricistronic rRNA transcript (SSU−rRNA, 5.8S rRNA, LSU−rRNA)
Gene
SAS10
MAK5
FCF1
UTP10
UTP20
REX2
SPB1
KRR1
RPL37B
LAS1
RRP5
RNH70
URB1
NOP14
RLP7
REX3
RAT1
CSL4
NOP58
MAK16
RMP1
DIP2
PNO1
NOP9
ROK1
YGR251W
RRP15
RRP43
UTP13
MTR4
ENP1
GRC3
RPS21B
MAK11
DIS3
UTP14
PWP2
RRP42
KRI1
UTP23
MRD1
RRP46
NSA2
RPL17B
RPF2
RRP6
RPL37A
RPS18B
RPL37B
LRP1
RPS21A
DBP3
RPS18A
RPS0A
RCL1
DBP10
TOM1
TIF6
ESF2
PRP43
YNR024W
DBP6
RRS1
NOC4
BUD23
BUD21
RPS0B
MTR3
LOC1
RAI1
Z_Shift_K_Gem
Z_Im_K_Gem
Z_Shift_K_Cyt
Z_Im_K_Cyt
Z_Shift_L_Gem
Z_Im_L_Gem
Z_Shift_L_Cyt
Z_Im_L_Cyt

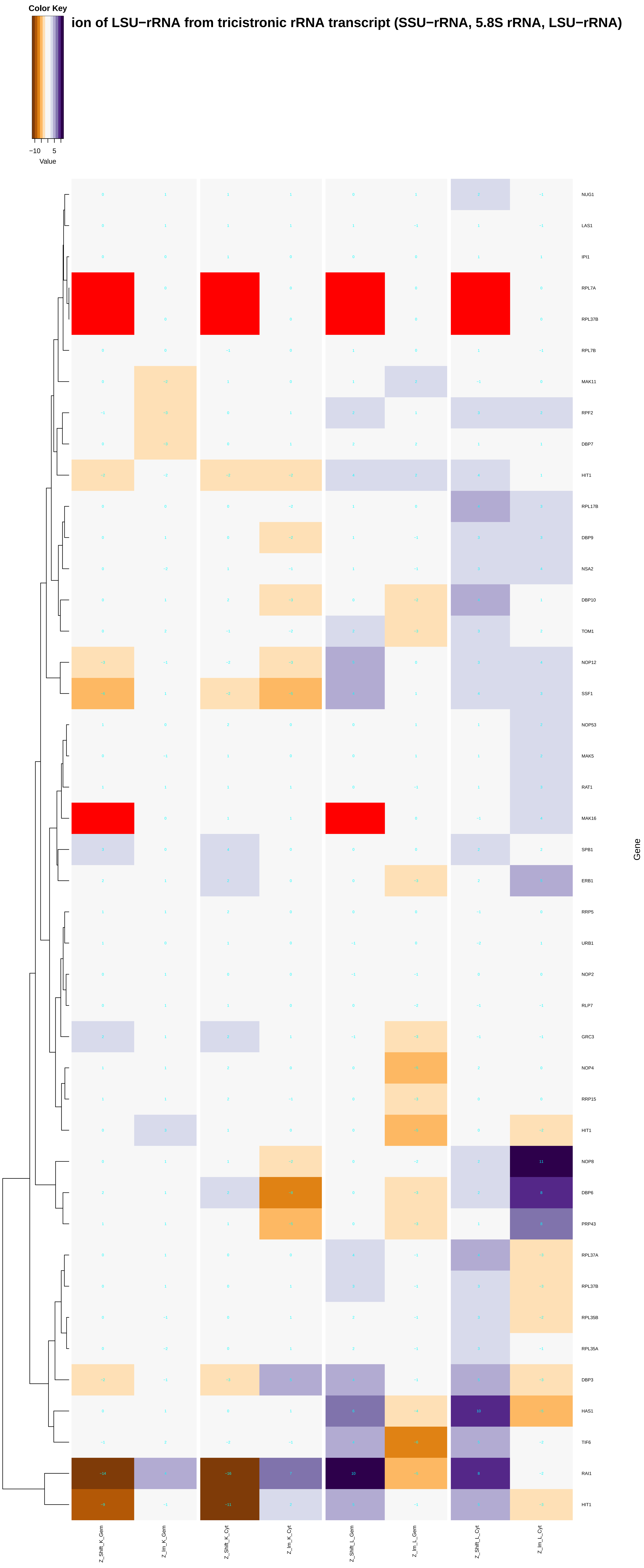

Color Key
−10 5
Value
ion of LSU−rRNA from tricistronic rRNA transcript (SSU−rRNA, 5.8S rRNA, LSU−rRNA)
NUG1
LAS1
IPI1
RPL7A
RPL37B
RPL7B
MAK11
RPF2
DBP7
HIT1
RPL17B
DBP9
NSA2
DBP10
TOM1
NOP12
SSF1
NOP53
MAK5
RAT1
MAK16
SPB1
ERB1
RRP5
URB1
NOP2
RLP7
GRC3
NOP4
RRP15
HIT1
NOP8
DBP6
PRP43
RPL37A
RPL37B
RPL35B
RPL35A
DBP3
HAS1
TIF6
RAI1
HIT1
Gene
Z_Shift_K_Gem
Z_Im_K_Gem
Z_Shift_K_Cyt
Z_Im_K_Cyt
Z_Shift_L_Gem
Z_Im_L_Gem
Z_Shift_L_Cyt
Z_Im_L_Cyt

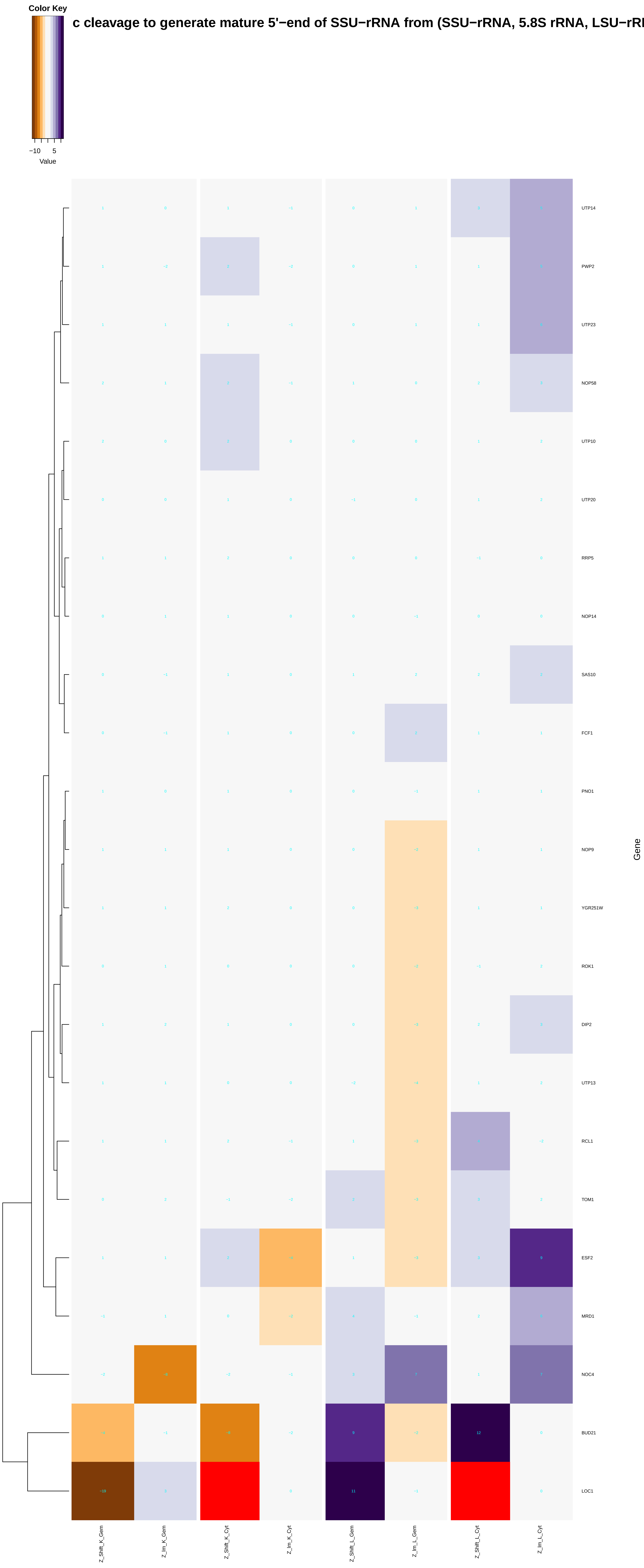
Color Key
−10
5
Value
c cleavage to generate mature 5'−end of SSU−rRNA from (SSU−rRNA, 5.8S rRNA, LSU−rR
UTP14
PWP2
UTP23
NOP58
UTP10
UTP20
RRP5
NOP14
SAS10
FCF1
PNO1
NOP9
YGR251W
ROK1
DIP2
UTP13
RCL1
TOM1
ESF2
MRD1
NOC4
BUD21
LOC1
Gene
Z_Shift_K_Gem
Z_Im_K_Gem
Z_Shift_K_Cyt
Z_Im_K_Cyt
Z_Shift_L_Gem
Z_Im_L_Gem
Z_Shift_L_Cyt
Z_Im_L_Cyt

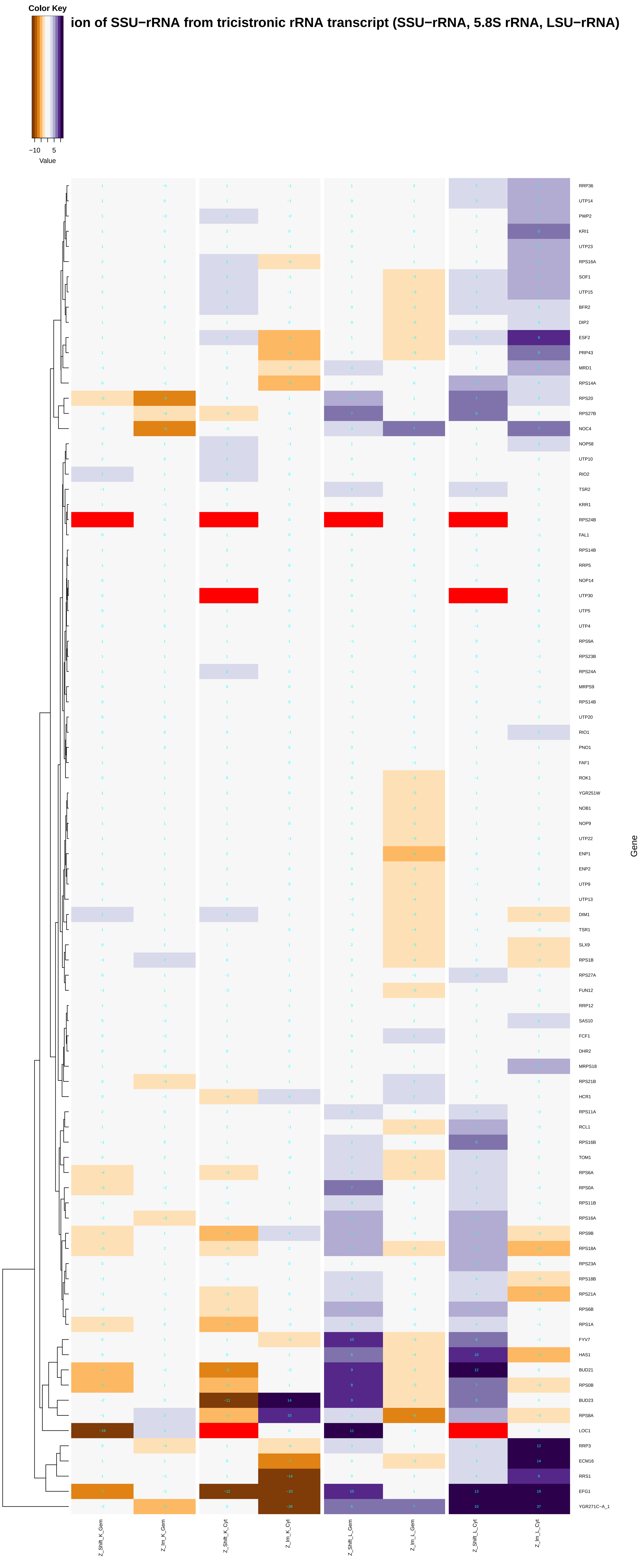
ion of SSU−rRNA from tricistronic rRNA transcript (SSU−rRNA, 5.8S rRNA, LSU−rRNA)
Color Key
−10
5
Value
RRP36
UTP14
PWP2
KRI1
UTP23
RPS16A
SOF1
UTP15
BFR2
DIP2
ESF2
PRP43
MRD1
RPS14A
RPS20
RPS27B
NOC4
NOP58
UTP10
RIO2
TSR2
KRR1
RPS24B
FAL1
RPS14B
RRP5
NOP14
UTP30
UTP5
UTP4
RPS9A
RPS23B
RPS24A
MRPS9
RPS14B
UTP20
RIO1
PNO1
FAF1
ROK1
YGR251W
NOB1
NOP9
UTP22
ENP1
ENP2
UTP9
UTP13
DIM1
TSR1
SLX9
RPS1B
RPS27A
FUN12
RRP12
SAS10
FCF1
DHR2
MRPS18
RPS21B
HCR1
RPS11A
RCL1
RPS16B
TOM1
RPS6A
RPS0A
RPS11B
RPS16A
RPS9B
RPS18A
RPS23A
RPS18B
RPS21A
RPS6B
RPS1A
FYV7
HAS1
BUD21
RPS0B
BUD23
RPS8A
LOC1
RRP3
ECM16
RRS1
EFG1
YGR271C−A_1
Gene
Z_Shift_K_Gem
Z_Im_K_Gem
Z_Shift_K_Cyt
Z_Im_K_Cyt
Z_Shift_L_Gem
Z_Im_L_Gem
Z_Shift_L_Cyt
Z_Im_L_Cyt

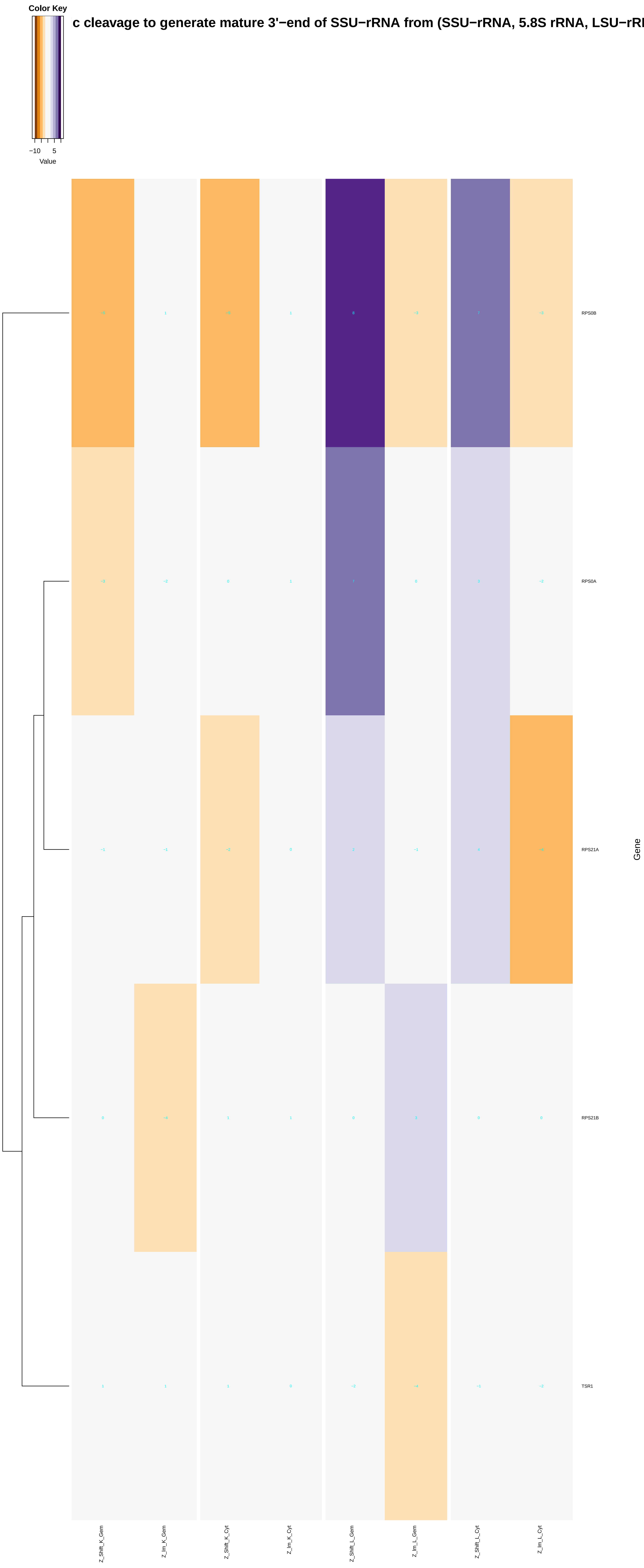
c cleavage to generate mature 3'−end of SSU−rRNA from (SSU−rRNA, 5.8S rRNA, LSU−rRI
Color Key
−10
5
Value
RPS0B
RPS0A
RPS21A
RPS21B
TSR1
Gene
Z_Shift_K_Gem
Z_Im_K_Gem
Z_Shift_K_Cyt
Z_Im_K_Cyt
Z_Shift_L_Gem
Z_Im_L_Gem
Z_Shift_L_Cyt
Z_Im_L_Cyt

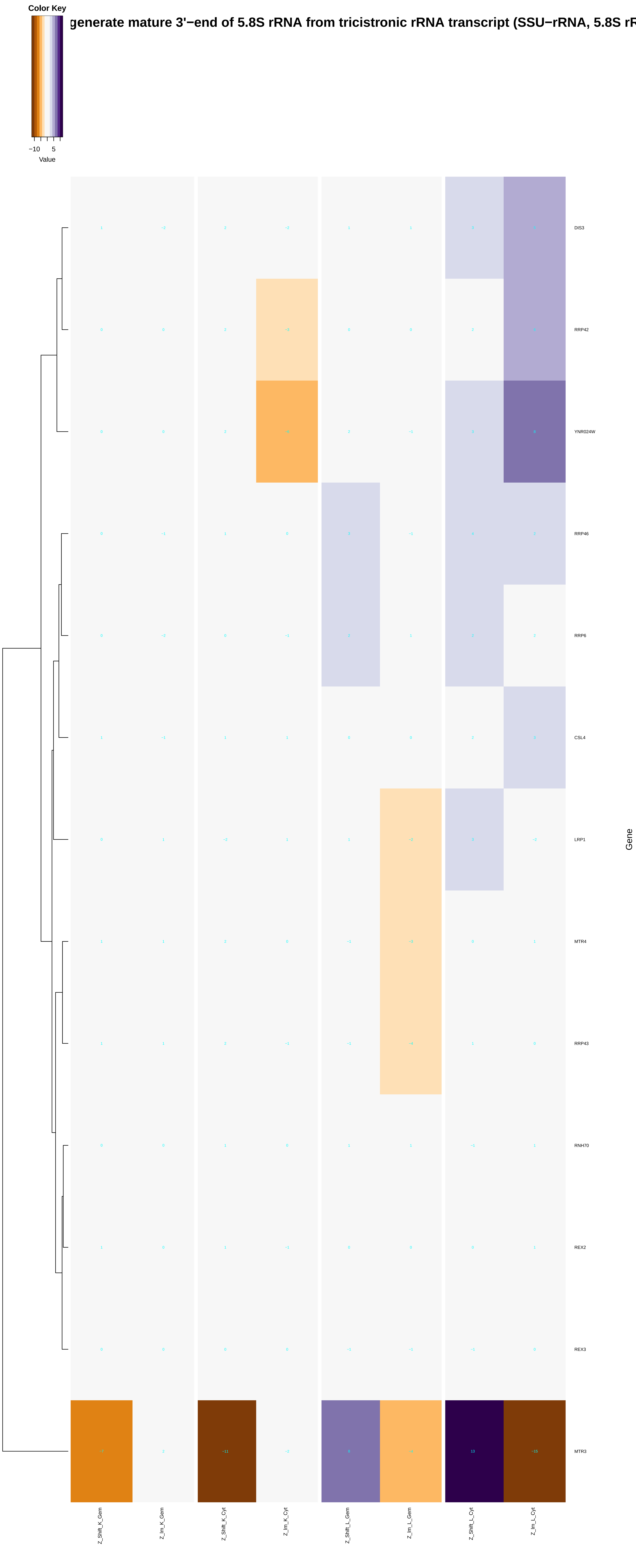
generate mature 3'−end of 5.8S rRNA from tricistronic rRNA transcript (SSU−rRNA, 5.8S rR
Color Key
−10
5
Value
DIS3
RRP42
YNR024W
RRP46
RRP6
CSL4
LRP1
MTR4
RRP43
RNH70
REX2
REX3
MTR3
Gene
Z_Shift_K_Gem
Z_Im_K_Gem
Z_Shift_K_Cyt
Z_Im_K_Cyt
Z_Shift_L_Gem
Z_Im_L_Gem
Z_Shift_L_Cyt
Z_Im_L_Cyt

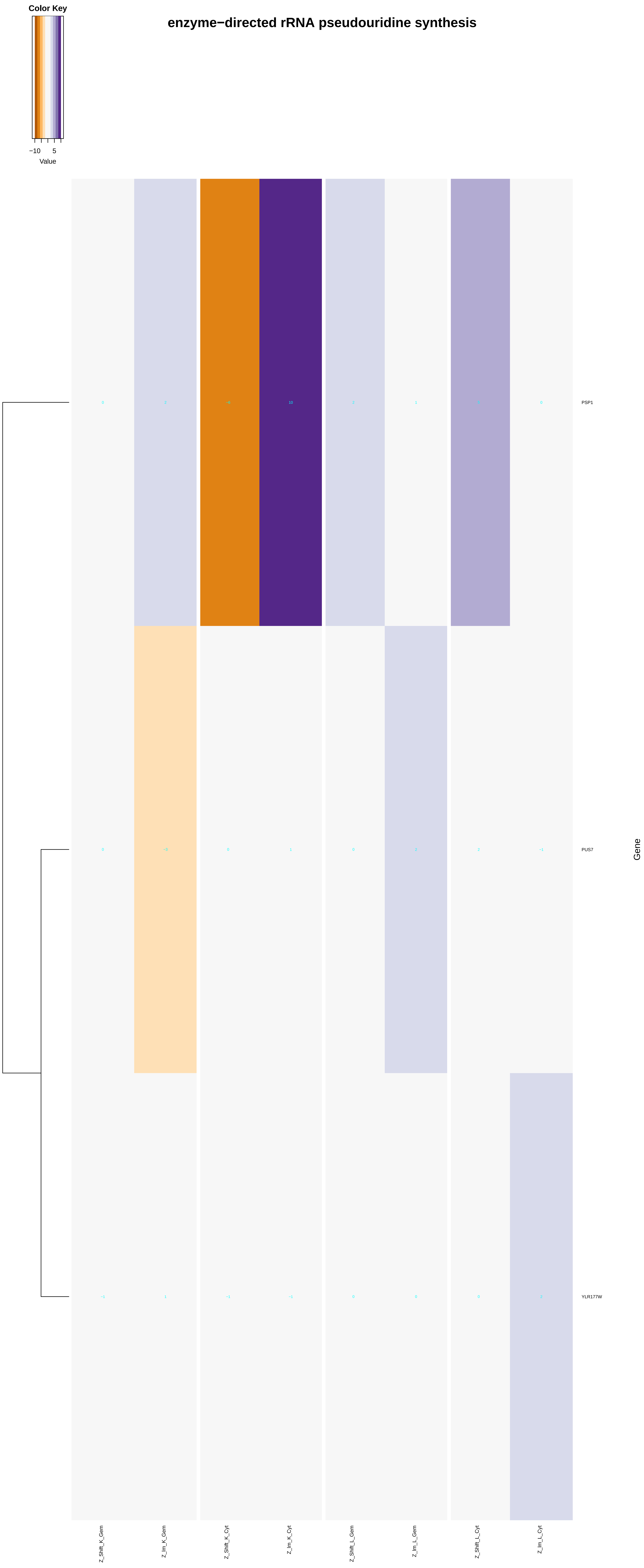
Color Key
−10
5
Value
enzyme−directed rRNA pseudouridine synthesis
0
2
−6
10
2
1
5
0
PSP1
0
−3
0
1
0
2
2
−1
PUS7
−1
1
−1
−1
0
0
0
2
YLR177W
Gene
Z_Shift_K_Gem
Z_lm_K_Gem
Z_Shift_K_Cyt
Z_lm_K_Cyt
Z_Shift_L_Gem
Z_lm_L_Gem
Z_Shift_L_Cyt
Z_lm_L_Cyt

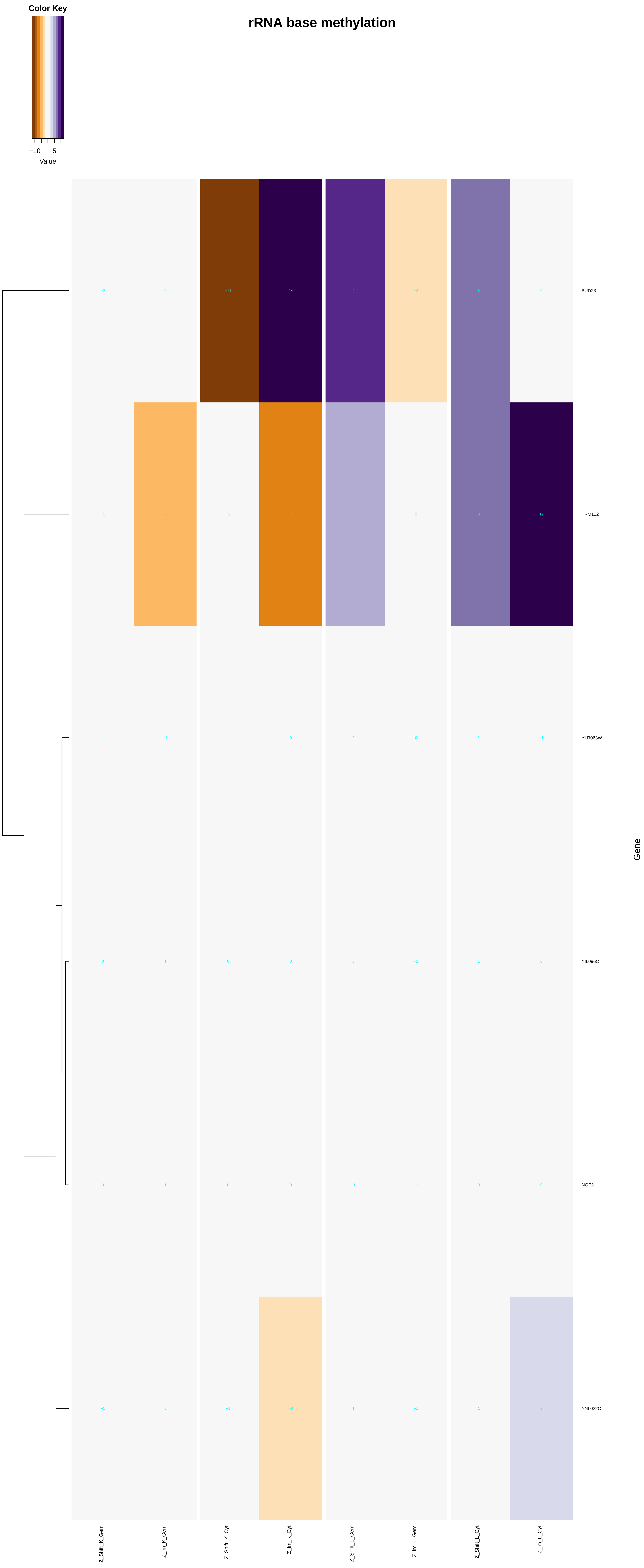

Color Key
−10
5
Value
rRNA base methylation
−2
0
−11
14
9
−2
8
0
BUD23
−1
−5
−2
−7
2
8
12
TRM112
1
−1
1
0
0
0
2
−1
YLR063W
0
1
0
0
0
−1
1
0
YIL096C
0
1
0
0
−1
−1
0
0
NOP2
−1
0
−1
−3
1
−1
1
2
YNL022C
Gene
Z_Shift_K_Gem
Z_Im_K_Gem
Z_Shift_K_Cyt
Z_Im_K_Cyt
Z_Shift_L_Gem
Z_Im_L_Gem
Z_Shift_L_Cyt
Z_Im_L_Cyt
